# Do Physicians Know Too Much?

**Published:** 1898-11

**Authors:** 


### *EDITORIAL NOTES AND COMMENTS.*

---

The Business office of THE JOURNAL is 305, 306 Fitten Building.

The Editorial office is Room 401, 402 Grand Opera House.

Address all Business communications to Dr. M. B. Hutchins, Mgr.

Make remittances payable to THE ATLANTA MEDICAL AND SURGICAL JOURNAL.

On matters pertaining to the Editorial and Original communications address Dr. Dunbar Roy, Grand Opera House, Atlanta.

Reprints of original articles will be furnished at cost price. Requests for the same should always be made *on the manuscript*.

We will present, post-paid, on request, to each contributor of an original article, twenty (20) marked copies of THE JOURNAL containing such article.

---

### DO PHYSICIANS KNOW TOO MUCH?

THE *Hospital*, published in London, has quite a lengthy editorial in its issue of August 20th, the substance of which might be well edited with the above caption. The writer takes a decidedly pessimistic view of the profession of medicine and bemoans the fact of the richness of the *materia medica* "which is annually poured out upon him by the manufacturing chemist." There is a great deal of truth in what he says, and what is true in Great Britain is still more so in the United States. For the benefit of our readers we quote what he says:

"The present writer has a grievance, a real, determined, angry grievance, against England, Germany, and America. These are the three countries which deluge medicine with physiology, good, bad, and indifferent, but mostly bad; which flood it with literature in the shape of medical books, with no soul of either science or practice in them, and which 'evolute' new remedies, not by the score, but by the thousand annually, not one of which in fifty is worth even so much as a second thought. The inevitable effect of all this upon the average minds in the profession is either to suffocate and so to paralyze them with what appears to be new knowledge, or else to so disgust the practitioner that he makes up his mind never to read at all, and on no earthly consideration whatever to experiment with a new drug. Medicine, in short, is swamped, drowned, stifled, and paralyzed by innumerable exploiters within and without its ranks; exploiters whose only object is the shortest possible cut, not to fame and fortune, but to notoriety and pelf. Now, all this has an exaggerated sound about it. But indeed and indeed, however exaggeratedly it sounds, it does not express one-tenth part of the miserable truth. The steady practitioner, whose aim is to supply his patients with the very best resources which the science of the times can afford, finds that about half his busy hours are spent in the brain-wearing, and what should be quite unnecessary, operation of separating the precious from the vile. And the vile is so very vile, and so overwhelmingly preponderant, that he almost wishes himself in the nether world, and permanently joined to the ranks of Sisyphus and Tantalus.

"And this is the reason: The profession is swamped with pedants; with persons in the consulting and special ranks who have a little money, no practice, and unlimited leisure; and these persons find their only consolation, the only salve of their disappointed self-love, in writing and reading all the rubbish which is annually poured out upon the profession, and so in persuading themselves that they are more learned and scientific than their better employed rivals. If it were not for the two or three thousand intolerable pedants in our ranks medical life would be worth living. As it is—well, a wise philosophy makes the best it can of the inevitable.

"For the strong, the mentally strong and resolute, there is, however, a remedy, even for so all-powerful a plague as the epidemic of medical books and new remedies. The strong have learnt, what all diligent stndents learn in time, the art of selection. They do not, and they will not, read the books and the papers of the exploiter, the pedant, and the notoriety-seeker. The study of a single page is generally quite sufficient to show what a book is made of. If it be pedantically expressed, if it be charged with a great show of learning, if it evinces a manifest anxiety to give the opinions of every other person, living or dead, who has ever written upon the same subject, then it is evident that it is a manufactured book. It is a pretty safe canon of literary criticism, especially of the medical order, that the book which gives publicity to everybody's opinion has no opinion of its own worth publishing. How much of other people's judgment did Lord Lister express when he was working out the antiseptic system of surgery and medicine? It was a frequent boast of the late Sir Andrew Clark, almost up to the time of his death, that he had 'never written a book.' What we need, almost more than anything else at the present moment in the medical profession, are two things: First, courageous independence of mind and judgment; and, secondly, a competent capacity for selection. Without these our practice has no rules, no certainty; it varies from day to day, and even from hour to hour; it is everywhere and it is nowhere. With them we shall daily place at the disposal of all our patients, if not the last new thing in drugs or the latest opinion in bacteriology, at least the best of the proved resources which the science of the times affords."

